# Two new species of *Fargesia* (Poaceae, Bambusoideae) from southwestern China

**DOI:** 10.3897/phytokeys.170.58780

**Published:** 2020-12-10

**Authors:** Xia-Ying Ye, Yu-Xiao Zhang, De-Zhu Li

**Affiliations:** 1 Agronomy and Life Science Department, Zhaotong University, Zhaotong, Yunnan 657000, China Kunming Institute of Botany, Chinese Academy of Sciences Kunming China; 2 Germplasm Bank of Wild Species, Kunming Institute of Botany, Chinese Academy of Sciences, Kunming, Yunnan 650201, China Zhaotong University Zhaotong China; 3 Yunnan Academy of Biodiversity, Southwest Forestry University, Kunming, Yunnan 650224, China Southwest Forestry University Kunming China

**Keywords:** *
Fargesia
*, new species, southwestern China, taxonomy, temperate woody bamboos

## Abstract

Two new species of *Fargesia*, one from Xizang (Tibet) and one from Yunnan, China, are described and illustrated. *Fargesia viridis* D.Z. Li & X.Y. Ye is characterized by its densely white powder, nearly solid internodes, yellow setose sheath scar and culm sheaths, and 4–6 leaves of large size. *Fargesia purpurea* D.Z. Li & X.Y. Ye has thinner culms (0.5–1.4 cm in diameter), a ring of 4–5 mm tall brown setae below nodes, fewer branches, glabrous sheath scar and culm sheaths, differentiated from the related species.

## Introduction

Tribe Arundinarieae, i.e. the temperate woody bamboos, is one of the three tribes of the subfamily Bambusoideae (Poaceae), containing approximately 581 species in 31 genera (Bamboo Phylogeny Group 2012; Clark et al. 2015; Clark and Oliveira 2018). These bamboos are distributed primarily in the temperate to subtropical zones of the Northern Hemisphere, with nearly 90% of species distributed in East Asia (Ohrnberger 1999; Li et al. 2006).

Among the 31 genera, *Fargesia* Franchet is the largest one, consisting of more than 90 species (Li et al. 2006; Yi et al. 2008), out of which, 85 species occur in China and 83 taxa are endemic to the country (Vorontsova et al. 2016). The *Fargesia* species are mainly distributed in temperate mountain areas (alt. 800–4300 m) of East Asia (Keng 1987; Yi 1988; Ohrnberger 1999; Li et al. 2006; Vorontsova et al. 2016). This group is especially common and diverse in the high elevation ecosystem of southwest China where they have undergone rapid diversification associated with the orogeny of the Hengduan mountains (Ye et al. 2019).

*Fargesia* is characterized by the presence of short-necked pachymorph rhizomes (usually < 20 cm), unicaespitose clumps, 7–15 branches at mid-culm nodes, semelauctant inflorescence, racemose to paniculate, compressed or open, with 3 stamens (Li et al. 2006). Although reproductive features are important for bamboo classification, vegetative morphological characters are usually used to distinguish species due to long flowering cycles (Janzen 1976; Zhang and Ren 2016). Based on morphological characters of buds and culm sheaths, Yi (1988) divided the genus *Fargesia* into two sections, F. sect. Ampullares Yi and F. sect. Fargesia (Keng and Wang 1996). The section Ampullares is distinguished by compound buds consisting of multiple distinct buds and deciduous culm sheaths. The section Fargesia is characterized by compound buds composed of several obscure buds and late deciduous or persistent culm sheaths, and contains four series, namely, ser. Murielae Yi, ser. Fargesia Yi, ser. Angustissimae Yi and ser. Yunnanenses Yi. The series *Murielae* has oblong or narrowly elliptical culm sheaths, with rounded apex, as wide as the base, while in the latter three series, the shape of culm sheaths is different and featured as narrowly triangular or narrowly orbicular-triangular, apex triangular or linear, much narrower than the base. Moreover, the texture and length of culm sheaths are varied in these three series. For example, the culm sheaths of ser. Fargesia and ser. Angustissimae are longer than internodes, but shorter or equal in ser. Yunnanenses. The culm sheaths of ser. Fargesia are apically leathery and narrowed for distal ca. 1/5 of length but apically thickly papery and narrowed for distal ca. 1/3–1/2 of length for species of ser. Angustissimae.

Although flowering is not frequent in this genus, it shows considerable diversity in vegetative morphology and many new species continue to be described (Yi 2000a, Yi 2000b, Yi 2000c; Yi et al. 2005, 2006; Yi et al. 2007; Yang and Yi 2013a, Yang and Yi 2013b) from southwest China. During floristic surveys of bamboos between 2015 and 2018, the authors collected vast specimens of *Fargesia* from southwest China. After scrutiny of the data available (Keng and Wang 1996; Li et al. 2006; Yi et al. 2008; Vorontsova et al. 2016), we found that several specimens could not be assigned to any described species. Here, we described two new species of *Fargesia* based on morphological comparison and the phylogenetic results (Ye et al. 2019).

## Materials and methods

Observation and measurement of morphological characters were undertaken using living plants in the field and specimens in the lab. Morphological features of related species were obtained from specimens and literature (Keng and Wang 1996; Li et al. 2006; Yi et al. 2008).

## Taxonomic treatment

### Fargesia viridis

Taxon classificationPlantaePoalesPoaceae

D.Z. Li & X.Y. Ye sp. nov.

9B44DC82-2E57-51AA-A68C-285F2DE0C403

urn:lsid:ipni.org:names:77213334-1

Figs 1 , 2

#### Diagnosis.

*Fargesia viridis* D.Z. Li & X.Y. Ye resembles *F. frigidis* Yi, *F. zayuensis* Yi and *F. similaris* Hsueh & Yi, but can be distinguished from *F. frigidis* by thinner and glabrous culm, more leaves on the ultimate branch, longer leaf sheath and large leaf blade, from *F. zayuensis* by shorter and thinner culm, solid internode, more leaves on the ultimate branch and broader leaf blade, and from *F. similaris* by solid internode, prominent sheath scar, setose culm sheath, glabrous petiole, more leaf number and larger leaf blade.

#### Type.

China, Yunnan, Gongshan County, along the road to Dulongjiang Town, 27°51'28"N, 98°26'46"E, 2667 m alt., 1 September 2015, *X.Y.Ye YXY272* (holotype & isotype: KUN!).

#### Description.

Rhizomes pachymorph, rhizome neck 3–6 cm long, 1–1.6 cm in diameter, solid. Culms 2–3 (4) m tall, pluricaespitose, 0.6–1.2 cm in diameter; internodes terete, 16–22 (30) cm long, densely white powdery and black when culms old, glabrous, nearly solid; nodes with weakly prominent supra-nodal ridge; sheath scar prominent, initially brown setose, with persistent remains of sheath base. Branches 8–10, fascicular, open; buds oblong, margins yellow-brown ciliolate. Culm sheaths persistent or tardily deciduous, leathery, narrowly rounded, 1/3 as long as internodes, yellow setose, densely at base and readily deciduous, longitudinal ribs prominent, margins yellow ciliolate, apex asymmetrical; auricles absent; oral setae absent or 1–2, ca. 2 mm long; ligule concave or truncate, ca. 1 mm tall, glabrous, fissured; blades erect or reflexed, linear-lanceolate, glabrous, narrower than the apex of culm sheath. Foliage leaves 4–6 per ultimate branch; sheath 3–4 cm long, glabrous, purple, margins ciliolate; auricles and oral setae absent; ligule truncate, ca. 1 mm tall; petiole 1–3 mm long, glabrous; blade lanceolate, 4–9 × 0.7–1.4 cm, glabrous, base broadly cuneate, secondary veins 2–3 pairs, transverse veins conspicuous, margins serrate. Inflorescence unknown.

#### Phenology.

New shoots July to August.

#### Etymology.

The specific epithet refers to the beautiful color of leaf blade.

#### Vernacular name.

Cuì Lǜ Jiàn Zhú (Chinese pronunciation); 翠绿箭竹 (Chinese name).

#### Distribution and habitat.

*Fargesia viridis* is only known from the type locality, the Dulongjiang Town. It occurs along the stream and grows as pure bamboo forest or under the evergreen broadleaved forest at an elevation of 2600–2800 m alt.

**Figure 1.**
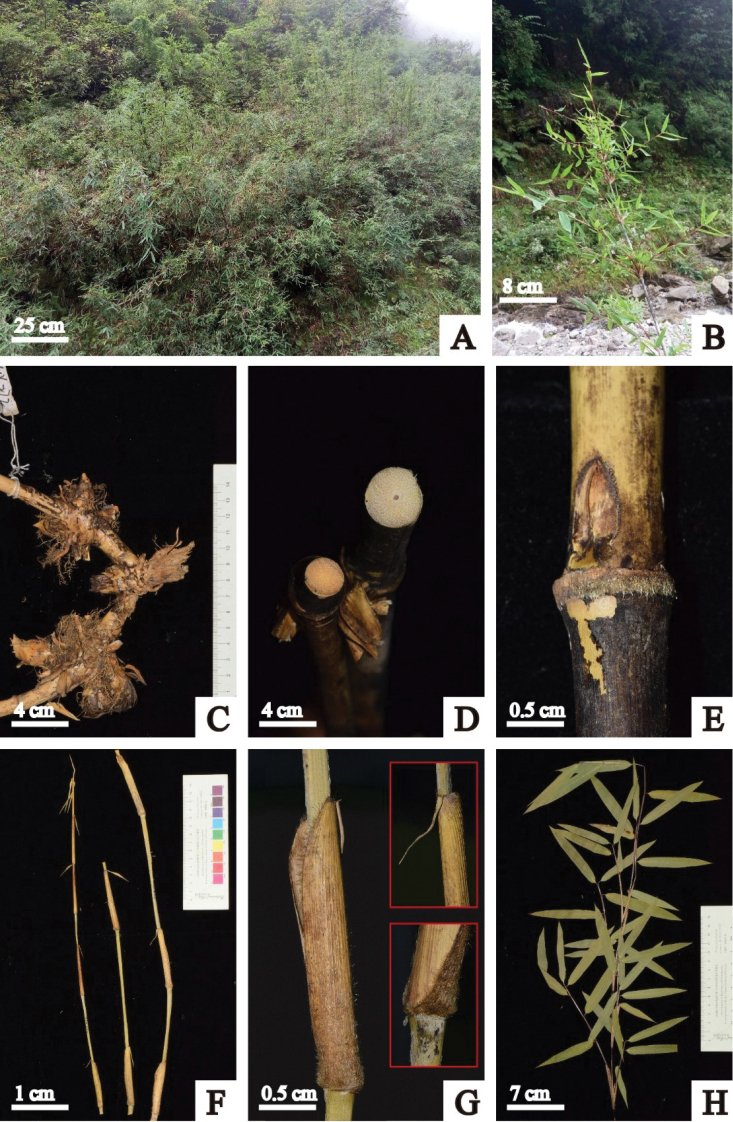
*Fargesia viridis* D.Z. Li & X.Y. Ye **A** habitat **B** individual **C** rhizome **D** culm showing solid and nearly solid internodes **E** culm bud and sheath scar with yellow setose **F** young culms with culm sheaths **G** culm sheath showing densely setose at base and oral characters **H** branchlet.

#### Notes.

Morphological comparisons between *Fargesia viridis* and the related species were provided in Table 1. Other four species of this genus were found in the Dulongjiang Town, i.e., *F. declivis* Yi, *F. sagittatinea* Yi, *F. acuticontracta* Yi and *F. praecipua* Yi, with this new species being easily distinguished from the other species in this region by its shorter and thinner culms, solid internodes (except *F. acuticontracta*), and shorter culm sheath (only 1/3 as long as internode).

**Table 1.** Morphological comparison of *Fargesia viridis* and its related species.

Characters	*Fargesia viridis*	*Fargesia frigidis*	*Fargesia zayuensis*	*Fargesia similaris*
Culm height	2–3 (4) m	1.5–3.5 m	6 m	Shrubby
Culm diameter	0.6–1.2 cm	1–1.7 cm	0.8–1.5 cm	0.8–1.2 cm
Internode	16–22 (30) cm long, densely white powdery, glabrous, nearly solid	22–24 cm long, initially densely white waxy and white-gray setose below nodes, glabrescent, nearly solid	25–35 cm long, initially sparsely white powdery; hollow, wall 1.5–2 mm thick	9.5–18.2 cm long, white or black powdery below nodes, wall 2–3 mm thick, cavity filled with lamellate pith
Branch complement	8–10	4–13	5–10	3–8(15)
Sheath scar	Prominent, initially yellow setose, with persistent remains of sheath base	Very prominent, woody	Prominent	Weakly prominent
Culm sheath	Persistent or tardily deciduous, yellow setose and densely at base, readily deciduous, longitudinal ribs prominent, margins yellow ciliolate, apex asymmetrical	Gradually deciduous to persistent, very sparsely appressed light yellow setulose, upper margins yellow-‐brown ciliolate initially, longitudinal ribs conspicuous, apex asymmetrical	Gradually deciduous, abaxially slightly gray-brown setulose, margins brown ciliolate or not	Glabrous, margins densely ciliolate, apex slightly white powdery
Culm sheath oral setae	Absent or 1–2, 2 mm long	Absent	Readily deciduous	Absent or 1–3
Culm sheath ligule	Concave or truncate, ca. 1 mm	Convex or truncate, 1–1.5 mm, glabrous	Truncate, ca. 1 mm	Truncate, ca. 1 mm
Culm sheath blade	Erect or reflexed, triangular or linear-lanceolate	Reflexed, readily deciduous, triangular to linear-lanceolate	Readily deciduous, reflexed, rarely erect, linear-lanceolate	Erect, triangular-conical, glabrous
Leaf number of the ultimate branch	4–6	1–4	1–3	2–4
Leaf sheath	3–4 cm long, glabrous	1.5–2 cm long, glabrous	3–4 cm, glabrous	Glabrous or with white pubescent margins
Leaf oral setae	Absent	Absent or sometimes few	Absent	2–6, 2–4 mm long, yellow-brown or gray
Leaf ligule	Truncate, ca. 1 mm	Inclined- truncate, ca. 0.4 mm	Truncate, glabrous	Truncate, ca. 1 mm
Petiole	1–3 mm long	1 mm long	1 mm long	Sparely gray-white pubescent
Leaf blade	4–9 × 0.7–1.4 cm, glabrous, secondary veins 2–3 pairs	2.3–5.2 × 0.45–0.7 cm, glabrous, secondary veins 2 or 3 pairs	5–8.5 × 0.4–0.6 cm, glabrous, secondary veins 2 pairs	1.3–6.5 × 0.4–0.6 cm, glabrous or abaxially white-gray pubescent, secondary veins 2- or 3 paired
Habitat	Along the stream or under the evergreen broadleaved forest at the altitude of 2600–2800 m, northwest, Yunnan.	On the shady slope of barren hills at 3100–3700 m, west Yunnan.	Under the *Pinus* or broadleaved forest, 2500–3000 m, Zayu, Xizang (Tibet).	Unknown, Yunnan

**Figure 2.**
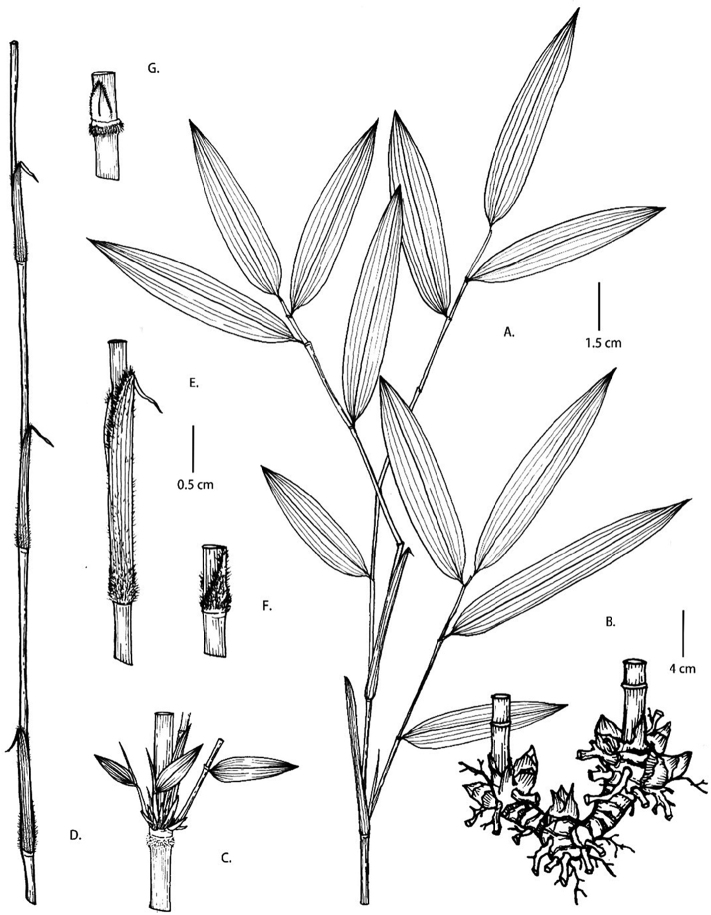
*Fargesia viridis* D.Z. Li & X.Y. Ye **A** branchlet **B** rhizome **C** node, showing branches and sheath scar with setose **D** yong culm with culm sheathes **E, F** culm leaves showing sheath and densely setose at base **G** culm buds.

### Fargesia purpurea

Taxon classificationPlantaePoalesPoaceae

D.Z. Li & X.Y. Ye sp. nov.

3D7049F6-1572-5E5E-AC13-AB1CB9CAC1FB

urn:lsid:ipni.org:names:77213335-1

Figs 3 , 4 , 5

#### Diagnosis.

*Fargesia purpurea* D.Z. Li & X.Y. Ye resembles *F. pauciflora* (Keng) Yi and *F. brevistipedis* Yi, but can be distinguished from the former by thinner and taller culms, a ring of 4–5 mm tall brown setae below nodes, glabrous sheath scar, fewer branches and more leaf number, from the latter by a ring of 4–5 mm tall brown setae below nodes, less branch number, glabrous sheath scar, oral setae absent and narrower leaf blade.

#### Type.

China, Xizang (Tibet), Zayu County, Xiachayu Town, bamboo mountain of new village, 28°31'14"N, 96°57'59"E, 2705 m alt., 24 August 2015, *X.Y.Ye & X.He YXY254-1* (holotype & isotype: KUN!).

#### Description.

Rhizomes pachymorph, rhizome neck 5–10 cm long, 1.2–2 cm in diameter, solid. Culms (3)4–5(6) m tall, unicaespitose, 0.5–1.4 cm in diameter; internodes terete, 30–46 cm long, white powdery and black when culms old, with a ring of 4–5 mm brown setae below nodes, longitudinal ribs prominent; wall 1–4 mm thick, cavity filled with lamellate pith; nodes with weakly prominent supra-nodal ridge; sheath scar prominent, with persistent remains of sheath base. Branches 3–7, open; buds triangular. Shoots purple, or with dark purple spots. Culm sheaths persistent, leathery, narrowly triangular, 1/3 as long as internodes, glabrous, longitudinal ribs prominent, upper margins ciliolate; auricles and oral setae absent; ligule truncate or inclined-truncate, 1–2 mm; blade reflexed, linear-lanceolate, glabrous, narrow than apex of culm sheath, readily deciduous. Foliage leaves 3–5 per ultimate branch; sheaths 2.5–4.5 cm long, glabrous, purple, margins ciliolate; auricles and oral setae absent; ligules truncate, ca. 1 mm; petiole 1–3 mm long; blades lanceolate, 5–12 × 0.5–1.4 cm, abaxially densely white pubescent, base cuneate, secondary veins 3–4 pairs, transverse veins conspicuous, margins serrate. Inflorescence unknown.

**Figure 3.**
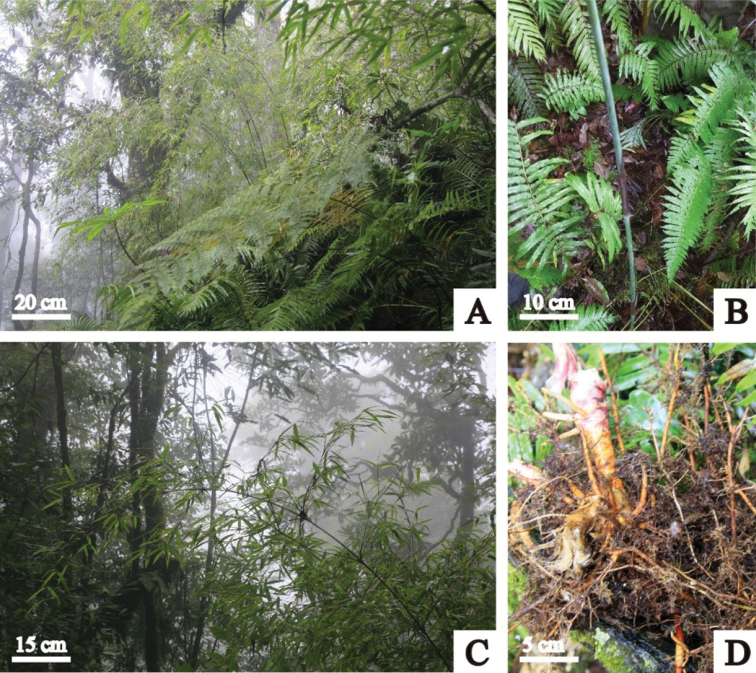
*Fargesia purpurea* D.Z. Li & X.Y. Ye **A** habitat **B** young and densely white powdery culm with purple culm sheath **C** individual **D** rhizome.

#### Phenology.

New shoots July to August.

#### Etymology.

The specific epithet refers to the color of culm sheath and leaf sheath.

#### Vernacular name.

Zǐ Qiào Jiàn Zhú (Chinese pronunciation); 紫鞘箭竹 (Chinese name).

#### Distribution and habitat.

*Fargesia purpurea* is only known from the type locality, bamboo mountain of new village in Zayu county. It grows under the evergreen broadleaved forest at an elevation of 2700–2800 m alt.

**Figure 4.**
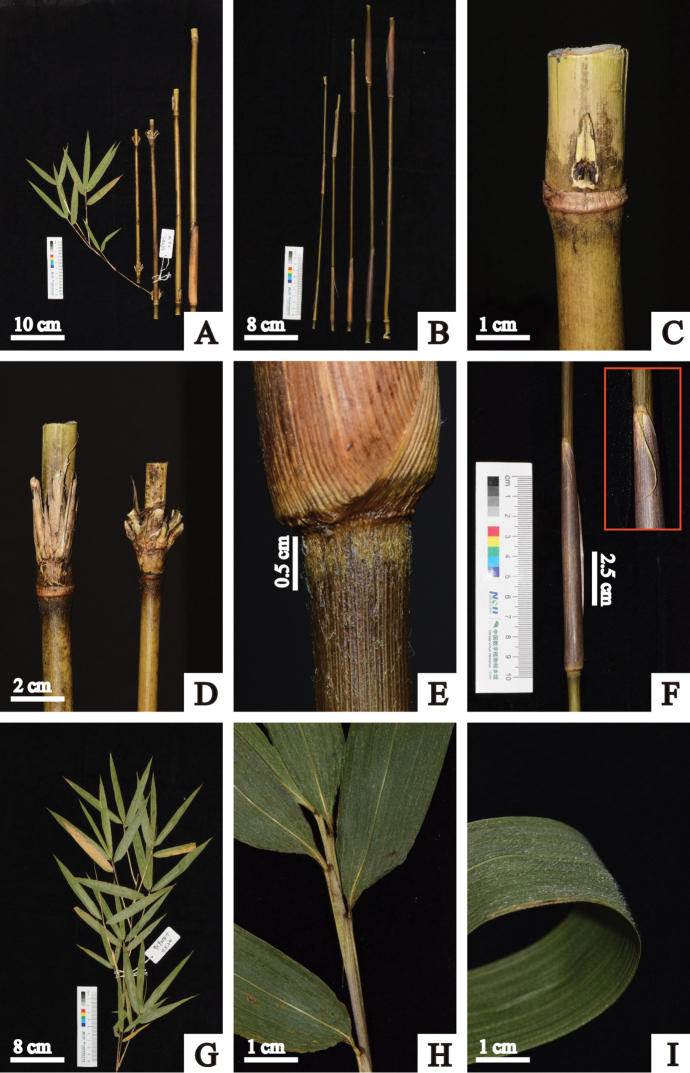
*Fargesia purpurea* D.Z. Li & X.Y. Ye **A** internodes, showing branches and persistent culm sheath **B** young culms with culm sheaths **C** culm bud **D** branches **E** node, showing brown setae below node **F** culm sheath, showing details of blade and ligule **G** branchlet **H** leaf sheath **I** abaxial surface of leaf, showing densely pubescence.

#### Additional specimens examined (paratype).

China, Xizang (Tibet), Zayu County, Xiachayu Town, bamboo mountain of new village, 28°32'04"N, 96°59'07"E, 2724 m alt., 24 August 2015, *X.Y.Ye & X.He YXY254‐2* (KUN!).

#### Notes.

Morphological comparisons between *Fargesia purpurea* and the related species were provided in Table 2. Two species of this genus were distributed in the Zayu county, namely, *F. zayuensis* Yi and *F. macclureana* (Bor) Stapleton, with this new species being easily distinguished from them in this region by its glabrous culm sheath and abaxially densely white pubescent leaf blade.

**Table 2.** Morphological comparison of *Fargesia purpurea* and its related species.

Characters	*Fargesia purpurea*	*Fargesia pauciflora*	*Fargesia brevistipedis*
Culm height	(3)4–5(6) m	2–4 m	4–5 m
Culm diameter	0.5–1.4 cm	1–3 cm	1.2–2 cm
Internode	30–46 cm long, longitudinal ribs prominent, densely white powdery, with a ring of 4–5 mm brown setulose; wall 1–4 mm thick	35–40 cm long, longitudinal ribs prominent, initially densely white powdery, glabrous; wall 2–3 mm thick	28‐35 (40) cm long, initially white powdery, glabrous; wall 1.5–2(3) mm thick
Branch complement	3–7	6–10	many
Sheath scar	Prominent, glabrous, with persistent remains of sheath base	Prominent, initially densely yellow-brown setose	Prominent, initially yellow-brown setose
Culm sheath	Persistent, glabrous, upper margins yellow-brown ciliolate initially	Persistent or gradually deciduous, glabrous or sparsely yellow-brown setose, margins brown ciliate	Persistent or gradually deciduous, glabrous or sparsely yellow-brown setose, white powdery, margins brown ciliate
Culm sheath ligule	Truncate or inclined-truncate, 1–2 mm	Truncate or arcuate, 1–2.5 mm	Truncate or arcuate, 1–1.5 mm
Culm sheath blade	Reflexed, readily deciduous,	Reflexed, linear-lanceolate, glabrous	Reflexed, linear or linear-triangular,
Leaf number of the ultimate branch	3–5	2 or 3	(3)5(6)
Leaf sheath	Purple, glabrous	Glabrous	Purple or light green, glabrous
Leaf oral setae	Absent	Absent	4–8, 5–6 mm long
Leaf ligule	Truncate, 1 mm tall	Arcuate or truncate, glabrous	0.5 mm tall
Petiole	1–3 mm long	Initially abaxially pubescent	Initially pubescent
Leaf blade	5–12 × 0.5–1.4 cm, secondary veins 3–4 pairs, abaxially densely pubescent	9–14 × 0.7–1.2 cm, secondary veins 2–4 pairs, abaxially pubescent	6.5–11.5 × 0.5–0.85 cm, secondary veins 3–4 pairs, initially abaxially gray pubescent
Habitat	Under the evergreen broadleaved forest at the altitude of 2700–2800 m, Zayu, Xizang (Tibet).	Under the *Pinus* or broadleaved forest, or under shrubs, 2000–3200 m, southwest Sichuan and northeast Yunnan.	Under shrubs at the elevation of about 1250 m, central Sichuan.

**Figure 5.**
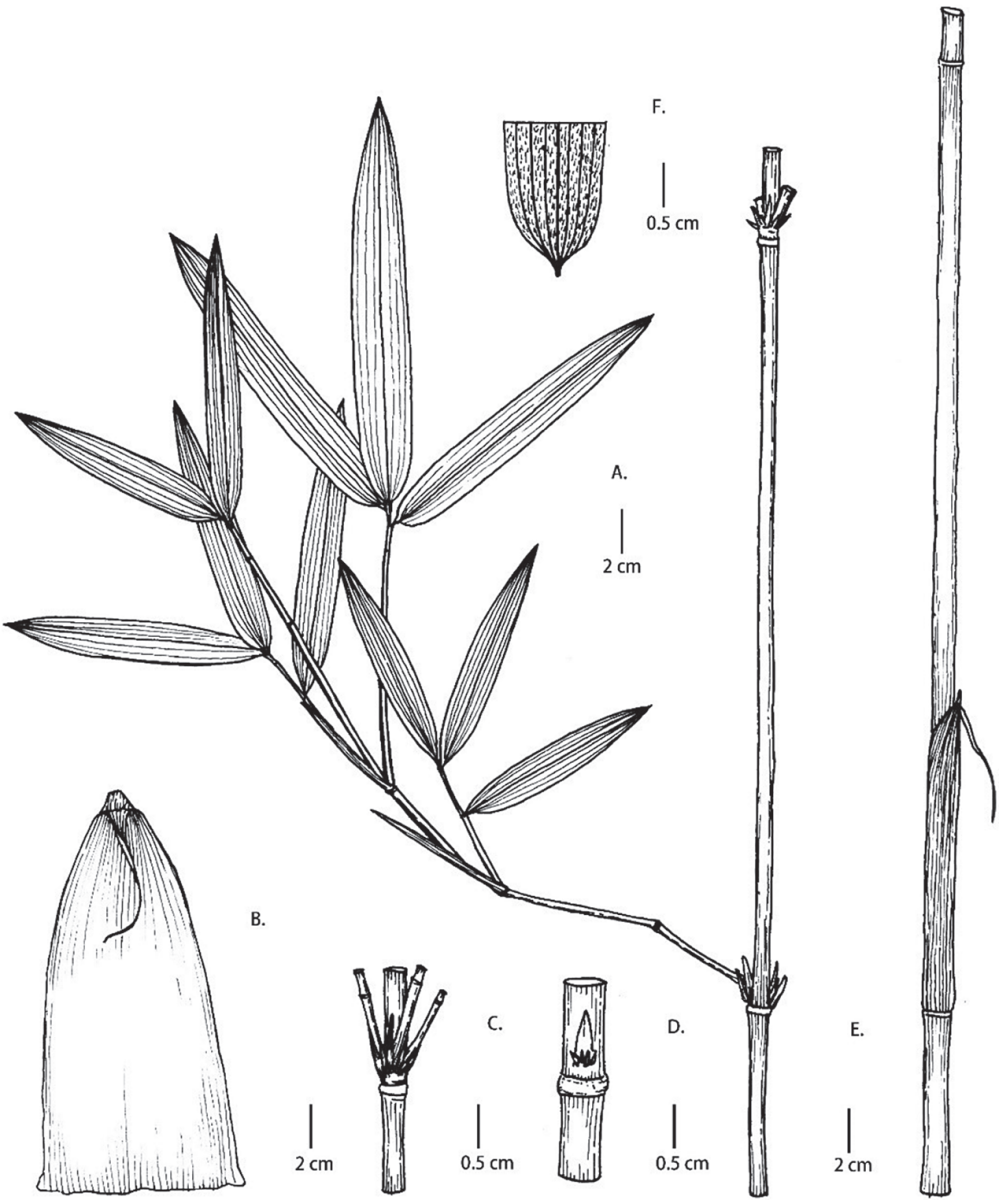
*Fargesia purpurea* D.Z. Li & X.Y. Ye **A** internode with branchlet **B** culm sheath abaxial view, showing culm leaf blade **C** node with branches **D** culm bud **E** yong culm with culm sheath **F** abaxial surface of leaf, showing densely pubescence.

## Discussion

Both *Fargesia viridis* and *F. purpurea* have persistent culm sheaths and buds containing several obscure buds, making them belong to the section Fargesia. The shape of culm sheaths is different from these two species. *F. viridis* is characterized by narrowly rounded culm sheath, with apex nearly as wide as base, which is similar to the species of the series *Murielae*. *F. purpurea* is characterized by triangular culm sheaths, shorter than internodes, with apex narrower than base; these features are similar to those species of the series *Yunnanenses*. Therefore, *F. viridis* and *F. purpurea* are assigned to the series *Murielae* and series *Yunnanenses*, respectively.

*Fargesia* is a polyphyletic genus and could be divided into three or four clades based on plastome sequences (Zhang et al. 2018; Zhou et al. 2019) and double-digested restriction enzyme-associated DNA sequencing (ddRAD-seq) data (Ye et al. 2019). *F. viridis* was classified as belonging to V-*Fargesia*4 clade based on the phylogenetic results of ddRAD-seq analyses (Ye et al. 2019), but no conclusion could be made for its position on the plastome phylogeny. Additionally, the phylogenetic relationship of *F. purpurea* in *Fargesia* has not been studied and that may be supplemented in the future.

*Fargesia viridis* (*F.* sp.2 in Fig. 2 of Ye et al. 2019) is closest to *F. frigidis* not only in morphology but also in phylogenetic relationships (Table 1, Ye et al. 2019), but the altitude distribution range of them are different. Moreover, *F. viridis* can be easily distinguished from *F. frigidis* by several morphologic characters, i.e. thinner culms, glabrous internodes, more leaves on ultimate branch. According to the identification keys, *F. viridis* is also similar to *F. zayuensis* and *F. similaris*; for example, they all have narrowly rounded culm sheath, with apex nearly as wide as the base, branch number usually above 5, auricles absent, glabrous leaf blade. However, a number of subtle features make *F. viridis* distinctive, such as internode nearly solid, densely white powdery culm, culm sheath persistent and densely yellow setose.

*Fargesia purpurea* resembles *F. pauciflora* and *F. brevistipedis* by its internode length, prominent sheath scar, culm sheath persistent, auricles and oral setae absent, and leaf blade abaxially pubescent, but differs in terms of the habitat, thinner culm, internode with a ring of 4–5 mm brown setulose, less branch number, glabrous culm sheath and sheath scar.

Mountains of Southwest China are the diversity center for *Fargesia* species; 80 out of 85 are distributed in this area and 73 of them are endemic. The two new species established here are also distributed in these mountains, indicating that the species diversity of *Fargesia* in this region may be beyond our knowledge. The species of *Fargesia* have an island-like distribution and allopatric speciation might have great impact on their diversity (Ye et al. 2019). However, the diversification of species could be caused by many reasons, such as heterogeneous environment, fluctuating climatic conditions, and adaptive evolution (Xing and Ree 2017; Ding et al. 2020). This genus with species distributed on a different elevation provides a case to disentangle the extrinsic and intrinsic factors that could promote species divergence. And research in this area may improve our ability to predict the evolutionary tendency and mitigate the threats posed by global warming to species distributed in the mountains of Southwest China.

## Supplementary Material

XML Treatment for Fargesia viridis

XML Treatment for Fargesia purpurea
